# Macrocyclic Lactones Differ in Interaction with Recombinant P-Glycoprotein 9 of the Parasitic Nematode *Cylicocylus elongatus* and Ketoconazole in a Yeast Growth Assay

**DOI:** 10.1371/journal.ppat.1004781

**Published:** 2015-04-07

**Authors:** Maximiliane Kaschny, Janina Demeler, I. Jana I. Janssen, Tetiana A. Kuzmina, Bruno Besognet, Theo Kanellos, Dominique Kerboeuf, Georg von Samson-Himmelstjerna, Jürgen Krücken

**Affiliations:** 1 Institute for Parasitology and Tropical Veterinary Medicine, Freie Universität Berlin, Berlin, Germany; 2 Schmalhausen Institute of Zoology, National Academy of Sciences of Ukraine, Kyiv, Ukraine; 3 Zoetis, Paris, France; 4 Zoetis International Services, Paris, France; 5 INRA, ISP, Animal Infection and Public Health, Nouzilly, France; Rush University Medical Center, UNITED STATES

## Abstract

Macrocyclic lactones (MLs) are widely used parasiticides against nematodes and arthropods, but resistance is frequently observed in parasitic nematodes of horses and livestock. Reports claiming resistance or decreased susceptibility in human nematodes are increasing. Since no target site directed ML resistance mechanisms have been identified, non-specific mechanisms were frequently implicated in ML resistance, including P-glycoproteins (Pgps, designated ABCB1 in vertebrates). Nematode genomes encode many different Pgps (e.g. 10 in the sheep parasite *Haemonchus contortus*). ML transport was shown for mammalian Pgps, Pgps on nematode egg shells, and very recently for Pgp-2 of *H*. *contortus*. Here, Pgp-9 from the equine parasite *Cylicocyclus elongatus* (Cyathostominae) was expressed in a *Saccharomyces cerevisiae* strain lacking seven endogenous efflux transporters. Pgp was detected on these yeasts by flow cytometry and chemiluminescence using the monoclonal antibody UIC2, which is specific for the active Pgp conformation. In a growth assay, Pgp-9 increased resistance to the fungicides ketoconazole, actinomycin D, valinomycin and daunorubicin, but not to the anthelmintic fungicide thiabendazole. Since no fungicidal activity has been described for MLs, their interaction with Pgp-9 was investigated in an assay involving two drugs: Yeasts were incubated with the highest ketoconazole concentration not affecting growth plus increasing concentrations of MLs to determine competition between or modulation of transport of both drugs. Already equimolar concentrations of ivermectin and eprinomectin inhibited growth, and at fourfold higher ML concentrations growth was virtually abolished. Selamectin and doramectin did not increase susceptibility to ketoconazole at all, although doramectin has been shown previously to strongly interact with human and canine Pgp. An intermediate interaction was observed for moxidectin. This was substantiated by increased binding of UIC2 antibodies in the presence of ivermectin, moxidectin, daunorubicin and ketoconazole but not selamectin. These results demonstrate direct effects of MLs on a recombinant nematode Pgp in an ML-specific manner.

## Author Summary

Macrocyclic lactones (MLs) are widely used drugs against parasitic nematodes, but drug resistance is rapidly increasing in prevalence and spatial distribution in parasites of ruminants and horses, and is suspected in human nematodes after mass drug applications. Changes in expression levels or the amino acid sequences of P-glycoprotein (Pgp) transporters have frequently been implicated in ML resistance, but direct evidence for transport of MLs by nematode Pgps is still missing. Here, cloning of pgp-9 of the equine parasite *Cylicocyclus elongatus* and its functional recombinant expression in a *Saccharomyces cerevisiae* yeast strain deficient in seven endogenous ABC transporters is described. Expression decreased susceptibility to several fungicidal mammalian Pgp substrates including e.g. actinomycin D and ketoconazole, but had no influence on susceptibility to the benzimidazole thiabendazole, which is active against both, yeasts and nematodes. Addition of some MLs strongly increased ketoconazole susceptibility in yeasts expressing *C*. *elongatus* Pgp-9, while other MLs had no effect. These interactions are a strong hint that some MLs act as substrates or at least as inhibitors of Pgp-9 mediated drug transport.

## Introduction

Due to their broad-spectrum antiparasitic activity with effects against both, nematodes and arthropods (endectocides), macrocyclic lactones (MLs) are among the most important antiparasitic drugs in veterinary and human medicine [1]. However, resistance to MLs is widespread in nematodes of small ruminants and currently increasing in prevalence and spatial distribution in nematodes of cattle, horses and humans [2–9] although for the latter the number of reports describing unresponsiveness of parasites to drugs is still only low and future investigations are required to formally prove resistance. In equines, ML resistance was initially observed in *Parascaris equorum* [10] but recently reports of ML resistant cyathostominae have also emerged [11–15].

In nematodes, the most important ML targets are glutamate-gated chloride channels (GluCl-Rs) whereas ionotropic γ-amino-butyric acid receptors respond only at higher drug concentrations [9].

Specific resistance mechanisms involving single nucleotide polymorphisms (SNPs) in the β-tubulin isotype 1 gene of nematodes from the order Strongylida are well known to be responsible for or at least strongly correlate with resistance to benzimidazoles (BZs) in ruminants and equines [16–20]. For levamisole resistance decreased density and open probability of nicotinic acetylcholine receptors, which are activated by levamisole, and certain splice variants encoding only truncated subunits of the receptors [21–23] have been described in resistant isolates. In contrast, no genotypes have been clearly involved in ML resistance except for a single report describing a SNP in a *Cooperia oncophora* GluCL-R subunit [24] but this specific change has never been observed in ML resistant nematodes in the field [25]. In the recent past, ABC (ATP-binding cassette) transporters and in particular P-glycoproteins (Pgps, i.e. orthologs of the mammalian ABCB1) have frequently been implicated in ML resistance mechanisms [26]. First hints that Pgps are involved in resistance were obtained by comparison of *pgp-2* alleles between ML susceptible and resistant isolates of *Haemonchus contortus* [27]. Using antibodies against a highly conserved epitope, larger amounts of active Pgps were detected on the egg shell of ML resistant *H*. *contortus* [26] and *C*. *oncophora* [28]. Moreover, the competitive Pgp inhibitor verapamil was shown to strongly sensitize non-parasitic stages of *C*. *oncophora*, whereas the verapamil effects on inhibition of development of the model nematode *Caenorhabditis elegans* in the presence of ivermectin (IVM) were only small [29]. Moderately increased efficacy of MLs in *C*. *elegans* strains which are deficient in individual Pgps was described in several assays [29–31]. Using *H*. *contortus* eggs it was also demonstrated that Pgps are activated by MLs [32]. Despite this large amount of work, direct evidence that an individual nematode Pgp is able to interact with MLs was missing for a long time. In the present study a *Saccharomyces cerevisiae* yeast strain deficient in seven endogenous ABC transporters [33] was used to express a recombinant Pgp-9 cloned from the equine parasitic nematode *Cylicocyclus elongatus* (Cyathostominae) and to compare interaction of several fungicidal Pgp substrates with this *Ceg*Pgp-9 in a simple and cheap yeast growth assay. Effects of different MLs were compared in the presence or absence of ketoconazole (Ket) to identify any potential competitive or enhancing effects of the drug combinations. Binding assays using the monoclonal antibody UIC2, which binds to a Pgp epitope only present during active transport, demonstrated activation of Pgp by MLs and fungicidal substances in the absence of a second drug.

## Materials and Methods

### Ethics statement

Adult *C*. *elongatus* were collected from euthanized naturally infected horses, which were bought from their owners. These animal experiments were in accordance with the “Tierschutzgesetz” in Germany and with the European Union directive 2010/63/EU. Experiments were approved by the Landesamt für Verbraucherschutz und Lebensmittelsicherheit (LAVES) in Hannover (Germany) under the reference number 06A435 and by the Landesamt für Gesundheit und Soziales (LaGeSo) in Berlin (Germany) under the reference number L 0088/10.

### Cloning of full-length Pgps

The full-length cDNA of *C*. *elongatus* pgp-9 was obtained using a strategy described previously [34]. Amplification of small fragments with degenerated primers was followed by rapid amplification of cDNA ends (RACE) PCR and amplification of full-length cDNAs. Initially, degenerated primers were designed based on sequence alignments of orthologous Pgp sequences of *C*. *elegans*, *Caenorhabditis briggsae*, *C*. *oncophora* and *H*. *contortus*. Oligonucleotide sequences are listed in S1 Table. RNA was extracted from adult nematodes and approximately 100 ng were reverse transcribed to cDNA according to the manufacturer’s protocol using random hexamer primers (Revert Aid First Strand cDNA Synthesis Kit, Thermo fisher Scientific). The PCR contained 16 μl H_2_O, 10 μM each forward and reverse primer, 2.5 μl Accu Prime buffer 1 (with dNTPs), 0.5 μl AccuPrime Taq DNA polymerase (Life Technologies) and 1 μl cDNA. PCR protocols were carried out as follows: After 2 min at 94°C for initial denaturation, 10 cycles of 94°C for 15 s, 50°C for 30 s and 68°C for 1 min were followed by 30 cycles with equal settings but an increased annealing temperature at 60°C. PCR fragments were gel-purified and cloned into pCR4-TOPO vector and sequenced by GATC Biotech (Konstanz).

RACE-PCR was carried out according to manufacturer’s instructions (3'/5'-RACE 2^nd^ generation Kit, Roche) as described recently [34] with primers and temperature profiles as listed in S1 Table. For 5'-RACE, cDNA synthesis was started with a gene-specific primer (S1 Table) and purified cDNAs were tailed with dATP to allow annealing of the oligo-dT anchor primer. PCR mixtures consisted of 18.75 μl H_2_O, 2.5 μl AccuPrime Buffer 1, 0.5 μM of each primer, 0.5 μl AccuPrime Taq DNA polymerase and 1.0 μl cDNA. PCR protocols for amplification were set as follows: Initial denaturation at 94°C for 2 min, followed by 40 cycles of 94°C for 15 s, 55°C for 30 s and 72°C for 1 min, and a terminal elongation at 72°C for 10 min.

For amplification of a full-length product, cDNA synthesis was carried out using oligo-(dT) primers. PCR mixture for amplification of *Ceg*pgp-9 contained 10 μl Q-solution (Qiagen), 0.5 μM each forward and reverse primer, 10 nM dNTPs, 0.5 μl Phusion II Hot Start Polymerase (Thermo fisher Scientific) and 4 μl cDNA in 50 μl 1×HF buffer. After initial denaturation at 98°C for 30 s, 40 cycles consisting of 98°C for 10 s, 68°C for 30 s and 72°C for 2 min were carried out followed by a terminal elongation at 72°C for 10 min.

### Phylogenetic analysis

Pgp protein sequences from nematodes as well as representative sequences from vertebrates, insects, mollusks and platyhelminthes were aligned using ClustalX2 [35]. The optimal amino acid substitution model was identified using Prottest 3.0 [36] with the number of evolution rate categories set to 8. Phylogenetic trees were calculated with PhyML 3.01 [37,38] assuming the same number of rate categories and the LG+I+F+G model [39] using both, nearest neighbor interchange (NNI) and subtree pruning and regraftment (SPR) moves. To avoid trapping of the iterative optimization process in a local maximum of the likelihood function, calculations started with one neighbor joining and five random trees. Branch support was obtained by conducting the Shimodaira-Hasegawa [SH] approximate likelihood ratio test and the Bayesian transformation of the approximate likelihood ratio test. Finally, the best tree was visualized using MEGA5 [40].

#### Expression of *C*. *elongatus* Pgp-9 in yeast

To re-amplify the open reading frame (ORF) of *C*. *elongatus* pgp-9 with and without stop codon, two sets of primer pairs were used. The reaction contained 41.8 μl H_2_O, 5 μl Accu Prime Buffer, 0.4 μM of each forward and reverse primer, 0.2 μl Accu Prime Taq DNA Polymerase High Fidelity (Life technologies) and approximately 80 ng plasmid DNA. After 2 min of initial denaturation at 94°C, 35 cycles of denaturation at 94°C for 15 s, annealing at 55°C for 30 s and elongation at 68°C for 4 min were performed. Terminal elongation was carried out at 68°C for 10 min. PCR products were ligated into the pYes2.1 TOPO vector and transformed into *E*. *coli* Top10 cells (Life technologies). After verification of the insert by sequencing, AD1234567 (AD1-7) yeast cells, which are deficient in seven endogenous efflux transporters [33], were transformed with purified plasmid DNA using the lithium acetate method according to the pYes2.1 TOPO manual. Transformed yeasts were identified after 48 h of incubation at 30°C on agar plates lacking uracil.

#### Analysis of transcription by RT-PCR

Yeast RNA was isolated from transformants selected for complementation of uracil auxotrophy using the Maxwell Simply RNA kit. After DNase digestion, cDNA synthesis with an oligo d(T) anchor primer was performed. Transcription was confirmed by RT-PCR using primers targeting fragments at the 3' end of the sequence, i.e. Ceg-Pgp-9-3R2 combined with either pYes-Pgp-9-lo or pYes-Pgp9-his (S1 Table). The reaction mixture contained 0.4 μM forward primer and 0.4 μM reverse primer either with or without stop codon, 2.5 μl Accu Primer buffer 1, 0.5 μl Accu Prime Taq DNA polymerase (Invitrogen), 18 μl bidest. H_2_O and 2 μl cDNA. An initial denaturation was performed at 94°C for 2 min followed by 35 cycles of denaturation at 94°C for 15 s, annealing at 55°C for 30 s and elongation at 72°C for 1 min. Correct PCR products were identified by fragment length after gel electrophoresis and sequence analysis.

#### Western blotting

SDS Page and transfer to nitrocellulose membranes were performed essentially as described previously [41]. The primary anti-V5 antibody (Life Technologies) was diluted 1:500 and the secondary goat anti-mouse antibody (Dianova) 1:5000. Detection was carried out using SuperSignal West Pico reagents (Thermo Scientific).

### Fluorescence activated cell scanning

Based on the monoclonal antibody UIC2, which is specific for a conserved epitope present in active Pgps, expression levels of *Ceg*Pgp-9 were determined by fluorescence activated cell scanning. Briefly, yeast cells were collected from overnight cultures by centrifugation and washed twice with PBS. After re-suspension in 3 ml PBS, three aliquots of 10 μl were transferred to 1.5 ml tubes and incubated with 1 ml blocking buffer (50 mg bovine serum albumin in 25 ml PBS) for saturation of non-specific binding sites. Samples were centrifuged and washed before incubation with the monoclonal antibody specific for active Pgps (UIC2, Drako), PBS or the isotypic antibody (mouse IgG 2aλ, clone HOPC-1, Beckman Coulter). Before flow cytometry analysis cells were again washed and filtered to remove cell agglomerates (30 μM mesh size). The cellular fluorescence intensities were measured with a MoFlo cytometer (Beckman Coulter). Instrument settings corresponded to the protocol as described elsewhere [42].

#### Growth assays with directly fungicidal drugs

Growth assays were carried out essentially as described elsewhere [43] with small modifications. In brief, *S*. *cerevisiae* cells (AD1-7*lac*Z, AD1-7*Ceg*Pgp-9 or AD1-7*Ceg*Pgp-9V5His) were grown overnight at 30°C and 250 rpm in 5 ml synthetic minimal medium (6.7 g/l yeast nitrogen base with amino acids, 1.92 g/l yeast drop-out medium supplement without uracil) supplemented with 2% galactose and 1% raffinose (SD+GR) to induce expression of transgenes. After counting in a Neubauer chamber, the required cell number was harvested by centrifugation and subsequently diluted in 1 ml SD+GR. Growth assays were performed in 96-well non-tissue culture treated, flat bottom plates (Becton Dickinson). Each well was inoculated with 4×10^4^ cells in 5 μl SD+GR and 95 μl SD+GR with 1% DMSO containing different drug concentrations. For Ket, final concentrations of 0.18 μM, 0.37 μM, 1.09 μM, 1.46 μM, 2.2 μM and 2.94 μM were used. Concentrations for actinomycin D, valinomycin and daunorubicin were 6.25 μM, 12.5 μM, 25 μM, 50 μM, 100 μM and 200 μM. Thiabendazol (TBZ) was used at final concentrations of 155 μM, 310 μM, 620 μM, 1240 μM and 2490 μM. The plate was closed and sealed with Parafilm M to avoid evaporation. During incubation over 48 h in a plate reader (Synergy 4, Biotech) at 30°C, plates were continuously shaken with a short delay before determination of absorption at 600 nm every 10 min to record growth curves. Cells incubated with medium containing 1% DMSO served as positive controls.

#### Growth assays with macrocyclic lactones

AD1-7*lacZ* and AD1-7*Ceg*Pgp-9V5His cells were cultured and processed as described above. Controls were prepared containing either no fungicide (both yeast strains) or 0.18 μM Ket (AD1-7*lacZ*) or 0.72 μM Ket (AD1-7*Ceg*Pgp-9V5His) in SD-GR with 1% DMSO. For identification of possible direct effects on yeast growth, all MLs were tested at 0.18, 0.36, 0.72, 1.44, 2.88 μM (AD1-7*lacZ*) and 0.72, 1.44, 2.88, 4.32, 5.76 μM (AD1-7*Ceg*Pgp-9V5His) without addition of Ket. To analyze any interaction of MLs with Ket in the presence of heterologous *Ceg*Pgp-9, cells were incubated with a combination of Ket and MLs at concentrations mentioned above. Plates were prepared and analyzed as described above.

#### Detection of changes in UIC2 binding in response to drugs

Yeast cells (AD1-7*lacZ* and AD1-7*Ceg*Pgp-9V5His) were obtained from fresh overnight cultures in SD+GR medium, washed once and then re-suspended in fresh SD+GR with 2% BSA at an OD_600_ of 0.5. Aliquots (200 μl) were shaken at 30°C and 250 rpm for 60 min before addition of drugs in 2 μl DMSO. Vehicle controls received only DMSO (1% final concentration as in all samples with drugs). Final concentrations for IVM, MOX and SLM were 4 μM and 1 μM while 2 and 0.5 μM were used for Ket and 200 μM and 6.25 μM for daunorubicin. After shaking at 30°C and 250 rpm for 10 min, the monoclonal antibodies UIC2 (recognizing active Pgp, LEAF purified anti-human CD243, Biolegend) and Mg2a-53 (IgG2a LEAF purified isotype control, Biolegend) were added in 2 μl to achieve a final concentration of 2 μg/ml. After further incubation with shaking at 30°C for 20 min, cells were rapidly cooled on ice, washed once with ice cold PBS and fixed at 4°C in 2.5% paraformaldehyde in PBS overnight. After two washes in PBS/2% BSA at room temperature, cells were suspended in secondary antibody solution (goat-anti mouse coupled to horseradish-peroxidase diluted 1:2000 in PBS/2% BSA) and shaken for 1 h at room temperature. After two wash steps with PBS, cells were re-suspended in 25 μl of solution I (containing stabilized H_2_O_2_) from the SuperSignal Pico West Pico chemiluminescence substrate (Thermo Scientific) and transferred to white, flat bottom 96 well plates. Then 25 μl of solution II (containing luminol) were added to each well. Plates were immediately transferred into a Synergy 4 (Biotek) plate reader and rapidly shaken at room temperature for 1 min. After 10 s delay, chemiluminescence signals were integrated over 1 s and recorded as relative light units.

#### Statistical analysis

Growth curves were fitted and statistically analyzed using the R software [44] with the grofit package [45] and GraphPad Prism 5.04 (GraphPad Software, San Diego, California). For comparing different yeast strains, relative growth rates were calculated by dividing the area under the curve (AUC) under certain experimental conditions to the mean AUC of the vehicle (negative control) with and without Ket, respectively. For calculation of concentration response curves, concentrations were log_10_ transformed. In order to include the negative controls despite log_10_ transformation of the drug concentrations, negative controls were set to 0.001 μM (Ket), 0.01 μM (actinomycin D, valinomycin and daunorubicin) or 10 μM (TBZ) before transformation. EC_50_-values of actinomycin D, valinomycin, daunorubicin, TBZ and Ket were calculated with a four parametric logistic regression analysis in GraphPad and due to previous normalization, the data were constraint to 1 as maximum value. Statistical differences in EC_50_ values were calculated with the extra sum of square F test implemented in GraphPad Prism. Direct effects of MLs on yeast growth were analyzed using a one way ANOVA with Bonferroni’s post hoc test to compare ML exposed samples to the no drug control. Differences and putative interactions between vehicles and ML concentrations with and without Ket were determined with a two way ANOVA and Bonferroni’s post hoc test.

Due to the low number of repeats (7–8) and unequal variances, non-parametric analysis was chosen to analyze data from UIC2 binding assays using chemiluminescence detection since normal distribution and equal variances as required for ANOVA analysis were not supported. Therefore, Kruskal-Wallis analyses followed by Bonferroni’s post hoc tests were conducted. First, AD1-7 expressing Pgp-9 incubated with UIC2 were compared with AD1-7Pgp-9V5His incubated with the isotype control and AD1-7*lacZ* with either UIC2 or the isotype control. In addition, all AD1-7 yeast samples with Pgp-9 expression incubated with drugs and UIC2 were compared to the DMSO control in the presence of the same antibody.

## Results

### Full length Pgps

**Fig 1:**
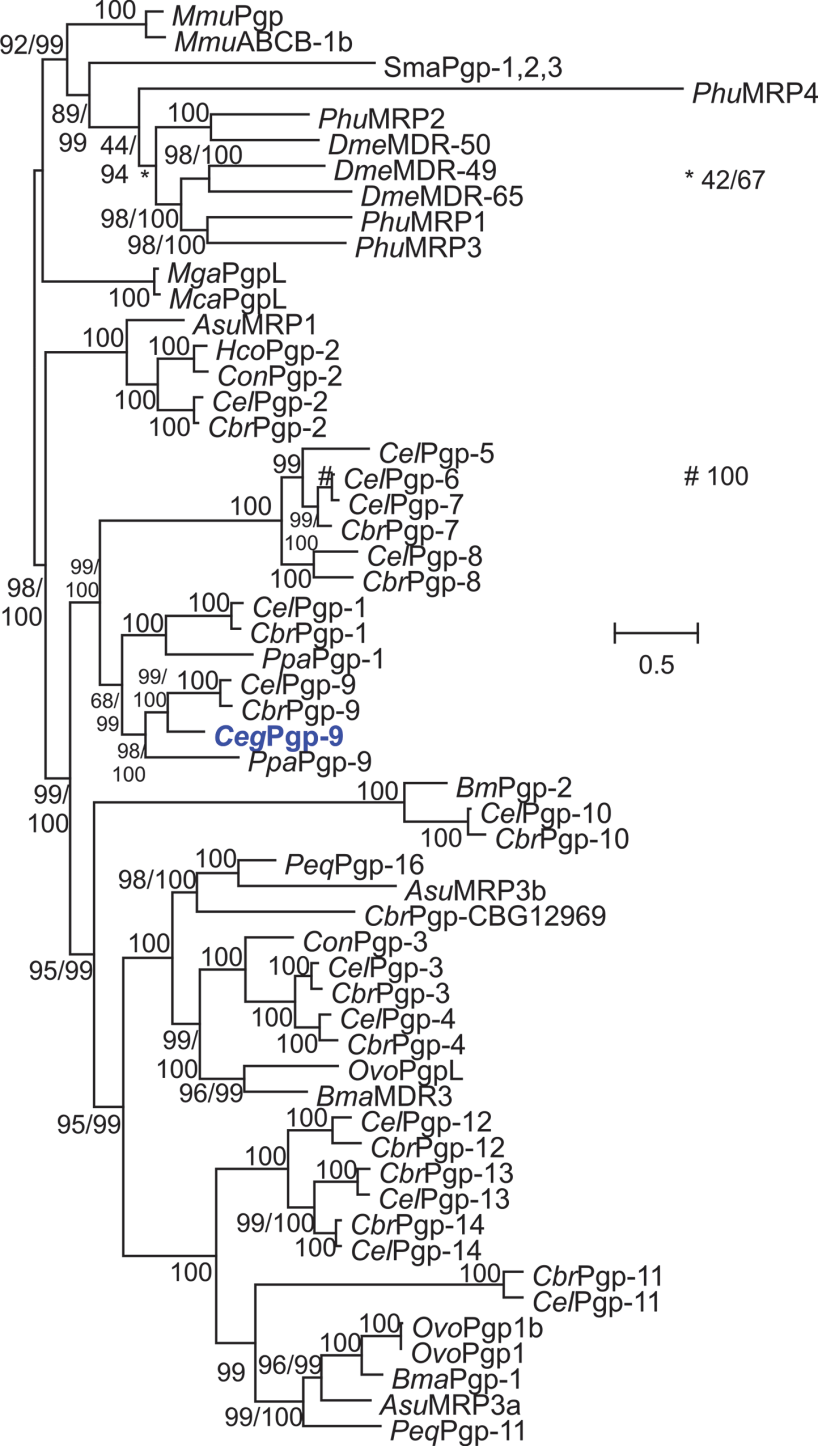
Phylogenetic analysis of nematode Pgps. The tree was calculated using the LG+I+F+G substitution model and PhyML 3.01. The number of amino acid substitution rate categories was set to eight. The results of the approximate likelihood ratio test modified according to Shimodaira and Hasegawa and of a Bayesian-like transformation of the approximate likelihood ratio test are provided before and after the slash, respectively. If only one value is given, the results of both tests were identical. As outgroup, Pgp sequences from mouse (*Mmu*), the insects *Drosophila melanogaster* (*Dme*) and *Pediculus humanus corporis* (*Phu*), the trematode *Schistosoma mansoni* (*Sma*) and the mussels *Mytilus californianus* (*Mca*) and *Mytilus galloprovincialis* (*Mga*) were used. The complete Pgp protein families of *Caenorhabditis elegans* (*Cel*) and *Caenorhabditis briggsae* (*Cbr*) were also included. Moreover, available annotated Pgp sequences of *Pristionchus pacificus* (*Ppa*) and of the parasites *Ascaris suum* (*Asu*), *Brugia malayi* (*Bma*), *Haemonchus contortus* (*Hco*), *Cooperia oncophora* (*Con*) and *Onchocerca volvolus* (*Ovo*) were included in the analysis. The new *C*. *elongatus* Pgp-9 sequence is highlighted in blue. Pgp, P-glycoprotein; MDR, multi-drug resistance protein. The scale bar represents the indicated number of substitutions per site. Accession numbers for protein sequences in the tree are provided in S2 Table.

After amplification of a small RT-PCR fragment using degenerated primers followed by 5' and 3' RACE PCR, amplicons containing the entire ORFs were obtained for *C*. *elongatus*. The fragments showed high similarity to *C*. *elegans* Pgp-9 as determined by Blast analyses. The cDNA sequence was deposited in GenBank under the accession no. KJ701410. The deduced amino acid sequence reveals the typical domain arrangement of Pgps with two similar halves, each consisting of an ABC transporter transmembrane domain (CDD accession number cd03249) containing six transmembrane helices followed by a nucleotide-binding domain (CDD accession number cl005249) containing the typical, highly conserved Walker A and B motifs as well as Q, D and H loops. Results of phylogenetic analysis using all Pgp proteins encoded in the genomes of *C*. *elegans* and *C*. *briggsae* as well as many previously published Pgps from other parasitic nematodes confirmed that the protein was a clear ortholog of *C*. *elegans* Pgp-9 and was therefore designated *C*. *elongatus* Pgp-9 (Fig 1).

### Recombinant expression of Pgp-9 in yeasts

**Fig 2:**
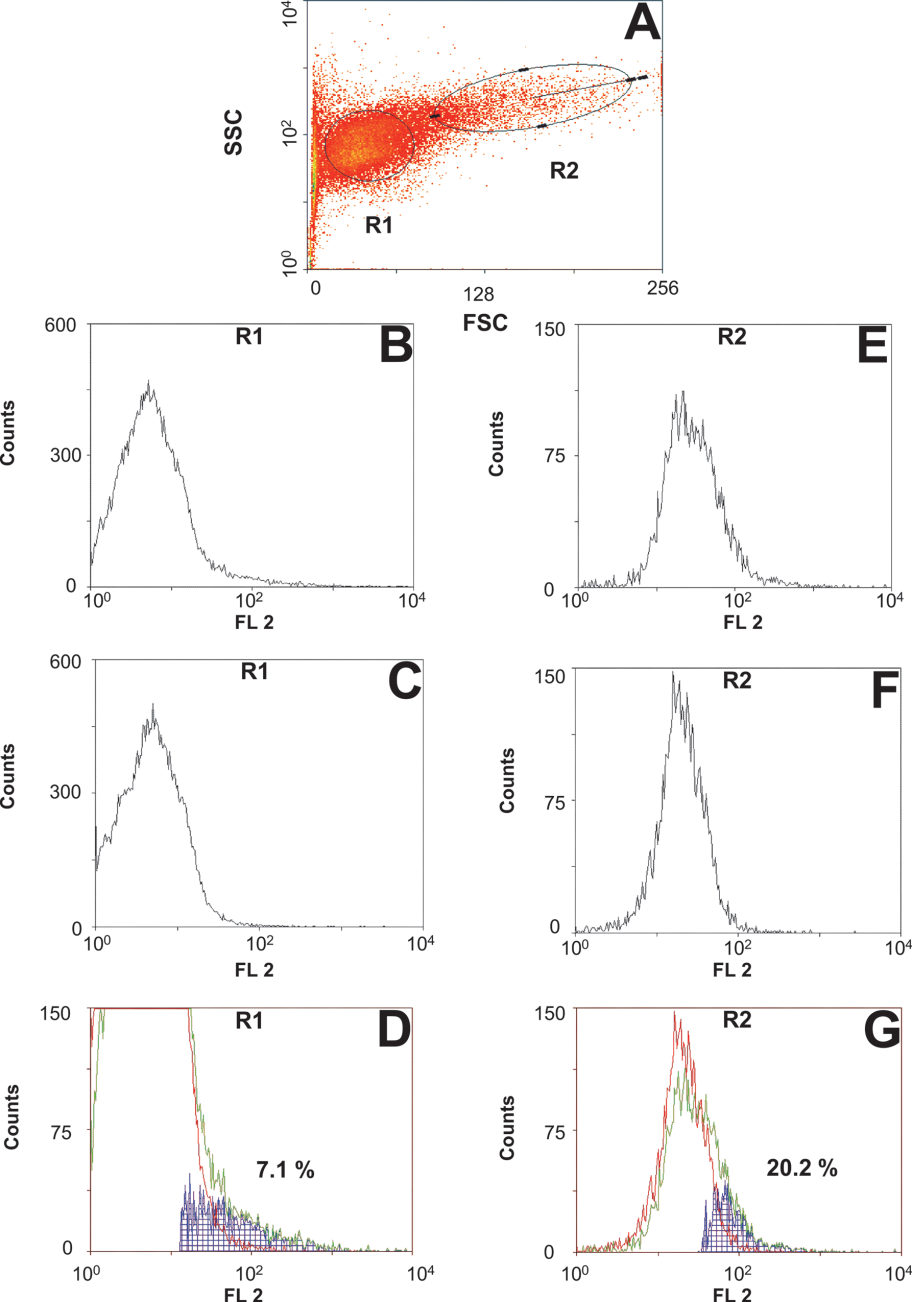
Flow cytometric analysis of Pgp expression on the surface of AD1-7*Ceg*Pgp-9V5His yeast cells. Cells were stained with the monoclonal antibody UIC2 binding to a highly conserved epitope exposed on active Pgps transporters. (A) Forward (FS) and side (SS) scatter dot plot of a representative sample. Small (R1) and large (R2) cells were analyzed independently for binding of UIC2. Histograms show cells from regions R1 (B, C) and R2 (E, F) stained either with the antibody recognizing active Pgp (UIC2) (B, E) or an isotype control (C, F). Histograms for the same region were overlaid and a subtraction of UIC2 stained minus isotype control stained samples is shown in blue for region R1 (D) and R2 (G).

The ORF of *C*. *elongatus* pgp-9 was amplified with and without stop codon to allow expression without and with a COOH-terminal tag (V5/6×His). PCR products were cloned into the pYes2.1 TOPO vector allowing galactose-induced expression. Plasmids were transformed into the *S*. *cerevisiae* strain AD1-7 which is deficient in seven major endogenous ABC transporters [33]. Initially, expression of Pgps in yeast was analyzed by RT-PCR and transcription of pgp-9 mRNAs could be confirmed (S1 Fig). Disappointingly, expression of *C*. *elongatus* Pgps in induced yeast cultures using Western blotting was not successful neither using the monoclonal antibody C219 (known to detect in Western blotting a highly conserved Pgp epitope which is present in *Ceg*Pgp-9) nor with the anti-V5 antibody although detection of β-galactosidase in the control strain was successful using the anti-V5 antibody (S2 Fig). FACS analysis using the monoclonal antibody UIC2, which is specific for a highly conserved epitope present on active Pgp transporters, revealed specific binding of the UIC2 antibody compared to the isotype control. Results of a representative experiment for cells expressing *Ceg*Pgp-9 are shown in Fig 2. According to forward (FS) and side (SS) scatter, two major populations of yeast cell were defined, i.e. small, non-granular (region R1) vs. large, granular (region R2) cells (Fig 2A). No obvious differences were found between cells expressing *Ceg*Pgp-9 with or without V5/6×His tag and the following data summarize experiments irrespective of the presence of a tag: In region R1, only a small fraction of the cells (4.4–8.9%) was positive in terms of increased binding of UIC2 in comparison to the isotype control (Fig 2B–2D). Despite much higher background fluorescence in region R2, a much higher number of the yeast cells in this region, i.e. 16.3–20.4%, had detectable amounts of active Pgp on their surface (Fig 2E–2G). No specific binding of UIC2 was detected for AD1-7*lacZ* yeasts, i.e. percentage of positive cells was between 0.23 and 0.6% (n = 3) in region R1 and 0.31 and 1.3% in region R2 (n = 3).

### Efficacy of fungicidal Pgp substrates and the fungicidal anthelmintic thiabendazole in a direct yeast growth assay

**Fig 3:**
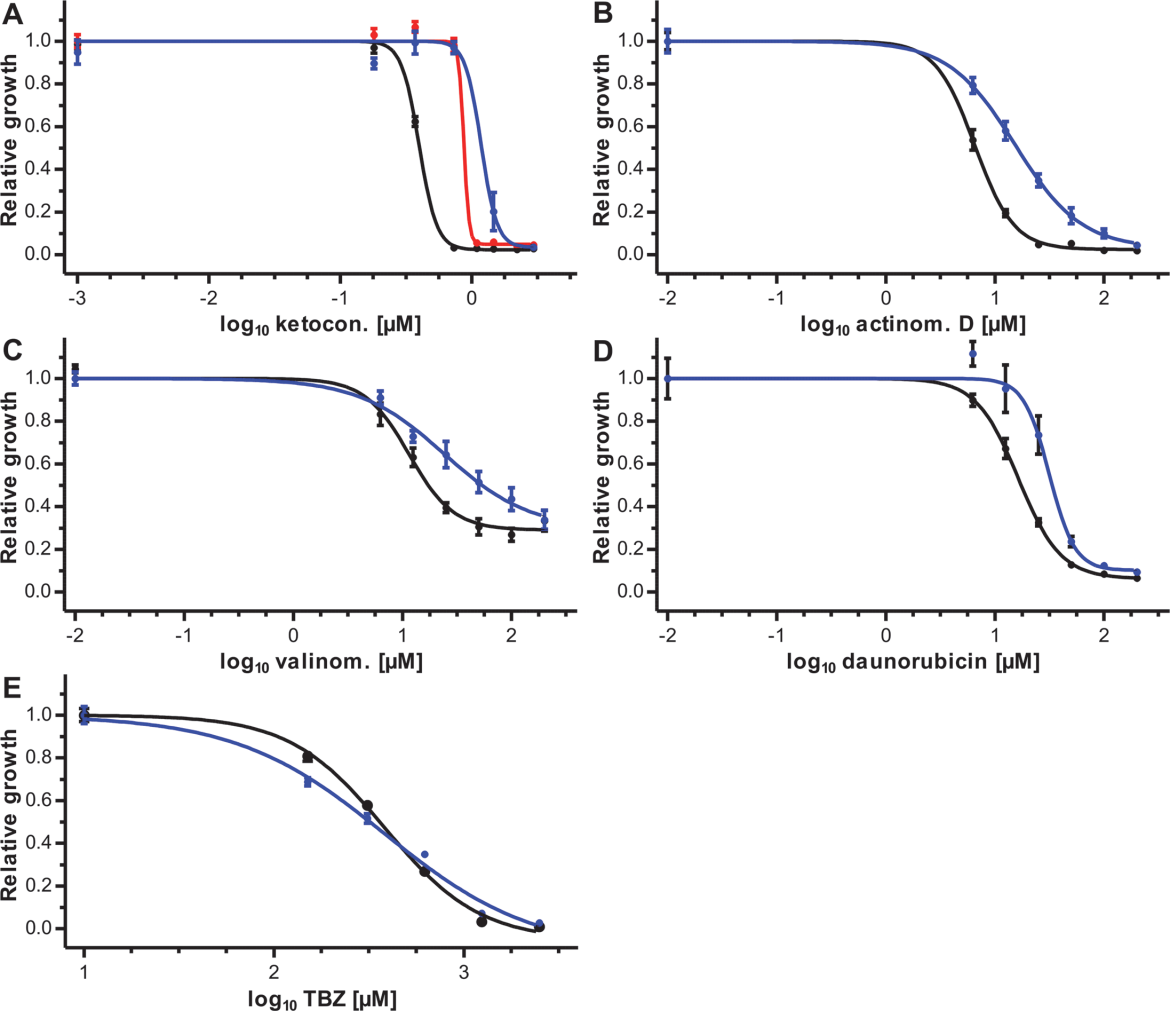
Effects of *Ceg*Pgp-9 expression on toxicity of directly fungicidal potential Pgp substrates. Effects of ketoconazole (A), actinomycin D (B), valinomycin (C), daunorubicin (D), and thiabendazole (E) were compared between the control yeast strain AD1-7 expressing *lacZ* (black) and AD1-7 strains expressing *Ceg*Pgp-9 either without tag (red) or with a V5/6×His tag (blue). Concentration response curves were recorded by measuring the OD_600_ every 10 min for 48 h. Data were retrieved from 18 data points per concentration (three plates each with six replicates) and normalized to the mean of the no drug control for the same strain (= relative growth). Mean relative growth (± SEM) were plotted against log_10_ of drug concentrations. Four parameter logistic regression curves were calculated and, due to the normalization, the upper asymptote was constrained to 1.

In order to demonstrate that the expression of recombinant Pgp-9 influences drug susceptibility of the yeast cells, the substrates of mammalian Pgps actinomycin D, daunorubicin, valinomycin and Ket were used [46,47]. For selected concentrations of TBZ as well as for a vehicle control typical growth curves are shown in S3 Fig as examples. Initial experiments were carried out using Ket and cells expressing Pgp-9 without tag (AD1-7*Ceg*Pgp-9) or with V5/6×His tag (AD1-7*Ceg*Pgp-9V5His), which were compared with cells expressing *lacZ* from the same vector backbone (AD1-7*lacZ*). *Ceg*Pgp-9 expression decreased susceptibility to Ket irrespectively of the presence of a V5/6×His tag (Fig 3A). The concentration response curves were extremely steep and even less than twofold differences in concentrations led to changes from ≥95% to ≤10% growth, in particular in the strain expressing *Ceg*Pgp-9 without a tag. Nevertheless, due to very small dilution steps, the EC_50_ values and confidence intervals of all three strains could be determined (Table 1). The EC_50_ values of the strains expressing *Ceg*Pgp-9 were approximately 2.2 (without tag) and 3.0 (with tag) fold increased. Although the difference between EC_50_ values of both strains was statistically not significant (p = 0.19), all further experiments were conducted using the AD1-7*Ceg*Pgp-9V5His strain. For actinomycin D, valinomycin and daunorubicin significantly higher EC_50_ values were observed when comparing AD1-7*Ceg*Pgp-9V5His strain with the control strain AD1-7*lacZ* (Fig 3B–3D, Table 1) with increases between 2.1 and 2.3 fold.

**Table 1:** EC_50_ values of AD1-7 yeast strains with or without expression of *C*. *elongatus* Pgp-9.

	AD1-7*lacZ*	AD1-7*Ceg*Pgp-9V5His	AD1-7*Ceg*Pgp-9	RR^b^
	EC_50_ [μM]	EC_50_ [μM]	EC_50_ [μM]	p value
	95% CI^a^	95% CI^a^	95% CI^a^	(vs. AD1-7*lacZ*)
	R^2^	R^2^	R^2^	
Ketoconazole	0.3959	1.176	0.8696	2.97^c^
	0.3809–0.4145	0.9857–1.402	0.6698–1.129	< 0.0001^c^
	0.9339	0.7713	0.9309	2.20^d^
				< 0.0001^d^
Actinomycin D	6.532	15.28	n.d.	2.34^c^
	6.022–7.086	12.54–18.62		<0.0001^c^
	0.9441	0.8890		
Valinomycin	11.48	24.18	n.d.	2.11^c^
	9.601–13.72	12.72–45.95		0.0031^c^
	0.7474	0.5998		
Daunorubicin	16.40	31.51	n.d.	2.11^c^
	15.15–17.75	25.81–38.49		<0.0001^c^
	0.9607	0.7538		
Thiabendazole	376.9	411.6	n.d.	1.09^c^
	343.2–413.9	308.6–549.1		0.4836^c^
	0.9721	0.9514		

^a^Confidence interval

^b^RR, resistance ratio

^c^AD1-7*Ceg*Pgp-9V5His vs. AD1-7*lacZ*

^d^AD1-7*Ceg*Pgp-9 vs. AD1-7*lacZ*

Since the anthelmintic TBZ is also an effective fungicide—although at relatively high concentrations—the ability of *Ceg*Pgp-9 to reduce susceptibility of AD1-7 yeast cells to TBZ could also be analyzed directly. Concentration response curves (Fig 3E) revealed only minimal differences between the control strain and the strain expressing *Ceg*Pgp-9 and EC_50_ values were virtually identical (Table 1).

### Effects of MLs in the absence and presence of ketoconazole in an indirect yeast growth assay

**Fig 4:**
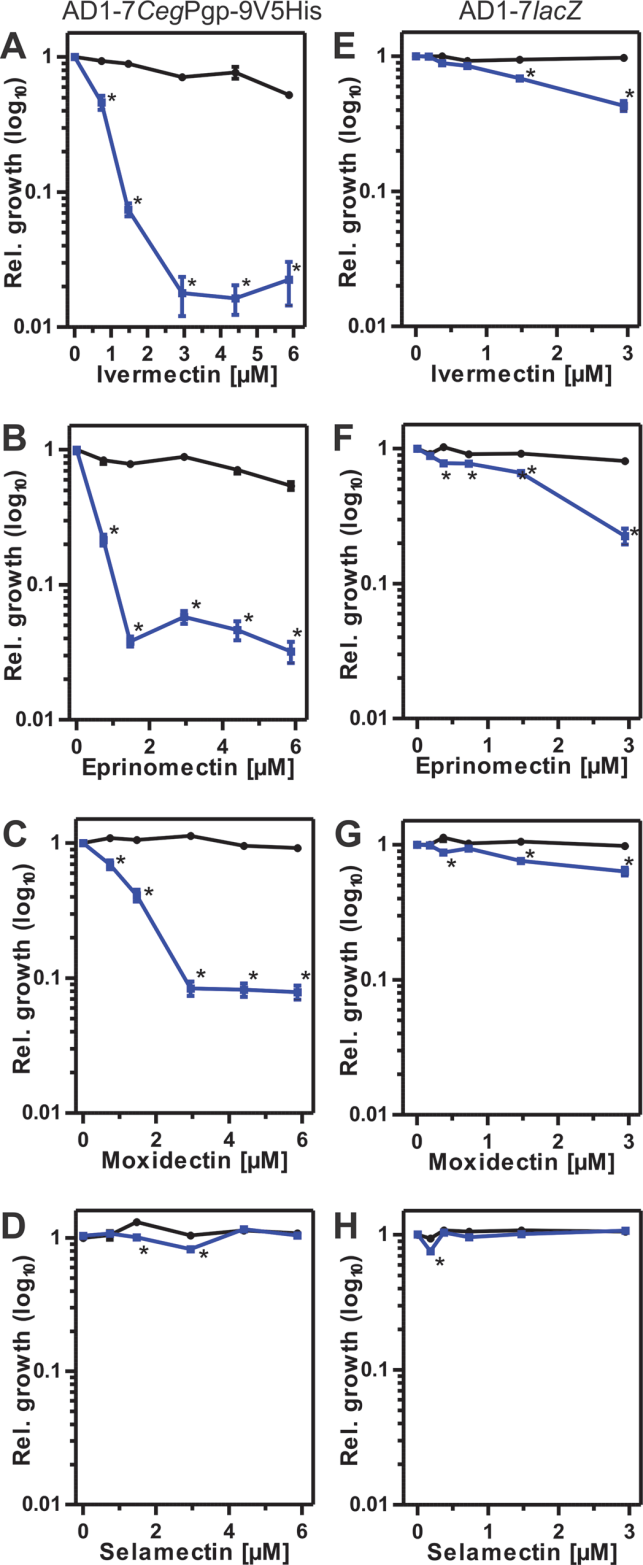
Effects of MLs on susceptibility to ketoconazole (Ket). Black symbols show relative growth in the absence and blue symbols in the presence of Ket. Data of 18 individual measurements were used. Concentrations of Ket were 0.72 μM for AD1-7*Ceg*V5His and 0.18 μM for AD1-7*lacZ*. The yeast strains AD1-7*Ceg*Pgp-9V5His (A-D) and AD1-7*lacZ* (E-H) were compared for effects of ivermectin (A, E), eprinomectin (B, F), moxidectin (C, G) and selamectin (D, H). A one-way ANOVA was conducted to test for effects of the macrocyclic lactone on yeast growth in comparison to the control without any drug (red *) and a two-way ANOVA was used to test for effects of macrocyclic lactone vs. the same drug concentration which depend on the presence of ketoconazole (black *). *, p < 0.05.

Since MLs were not known to exert any significant fungicidal effects, an indirect method was used to quantify ML interactions with *Ceg*Pgp-9. For this purpose, a critical concentration (*i*.*e*. the highest concentration showing no significant effect on yeast growth) of the fungicidal Pgp substrate Ket was used. For AD1-7*lacZ* this was 0.18 μM while AD1-7*Ceg*Pgp-9V5His still grew normally at 0.72 μM. MLs were then used in a concentration range starting with equimolar concentrations of Ket and ML and ending with up to 16 fold higher ML concentrations (Fig 4). These assays were conducted with the aim to test whether MLs can inhibit transport of Ket by *Ceg*Pgp-9, which would lead to increased susceptibility to Ket.

In the absences of Ket, high concentrations of IVM and eprinomectin (EPM) surprisingly showed a direct effect of the ML on growth of AD1-7*Ceg*Pgp-9V5His (Fig 4A and 4B). In the same strain, no significant direct concentration dependent effects of moxidectin (MOX), selamectin (SLM) and doramectin (DRM) were observed (Fig 4D and 4E and S4A Fig). With the exception of EPM, which significantly reduced growth at the highest concentration of 2.94 μM, there were no directs effects on growth of AD1-7*lacZ* (Fig 4E–4H and S4B Fig), which can be explained by the fact that ML concentrations used in this strain were generally lower.

When used in combination with Ket, even equal concentrations of EPM, IVM and MOX (but not of SLM and DRM) with Ket were able to significantly enhance the fungicidal effects of Ket in *Ceg*Pgp-9 expressing yeast cells (Fig 4A–4C, S4A Fig). Effects ranged between 31 and 79% inhibition of relative growth with the strongest effects exerted by EPM and the least pronounced effects by MOX. Twofold higher ML concentrations virtually abolished relative growth in the presence of EPM, allowing only minimal growth in the presence of IVM and reducing growth in the presence of MOX by approximately 59%. Remarkably, even at higher concentrations MOX only inhibited growth of AD1-7*Ceg*Pgp-9V5His by about 92% whereas maximal inhibition of growth in the presence of IVM and EPM was approximately 95% and 98%. In contrast to EPM, IVM and MOX, no effects were observed for SLM and DRM even at the highest concentrations used (Fig 4D, S4A Fig).

In contrast, effects of MLs on growth of AD1-7*lacZ* cells were much smaller and at eightfold higher concentrations of IVM, EPM or DRM than those of Ket, inhibition rates in the rage of 24–34% were observed (Fig 4E–4H, S4B Fig). Even 16-fold excess of MLs over Ket caused only 47–78% growth inhibition.

### Influence of drugs on the ability to bind the UIC2 antibody

Since UIC2 is known to bind only to active Pgps, binding of the antibody should increase in the presence of substrates. AD1-7*lacZ* and AD1-7*Ceg*Pgp-9V5His cells were incubated with drugs followed by addition of UIC2 or an isotype control and detection via a HRP-labeled secondary antibody and chemiluminescence. Since this type of assay is much more complicated in terms of sample handling than the growth assay, only selected drugs and two concentrations per drug were analyzed. Drug concentrations were chosen to have one concentration with a strong and one with no or only a minimal effect in the growth assay. For comparisons of MLs, one with a strong effect (IVM), one with a moderate effect (MOX) and one without effect (SLM) on growth in the presence of Ket were chosen. The same ML concentrations were used for all MLs and concentration with small and strong effects of IVM were selected.

**Fig 5:**
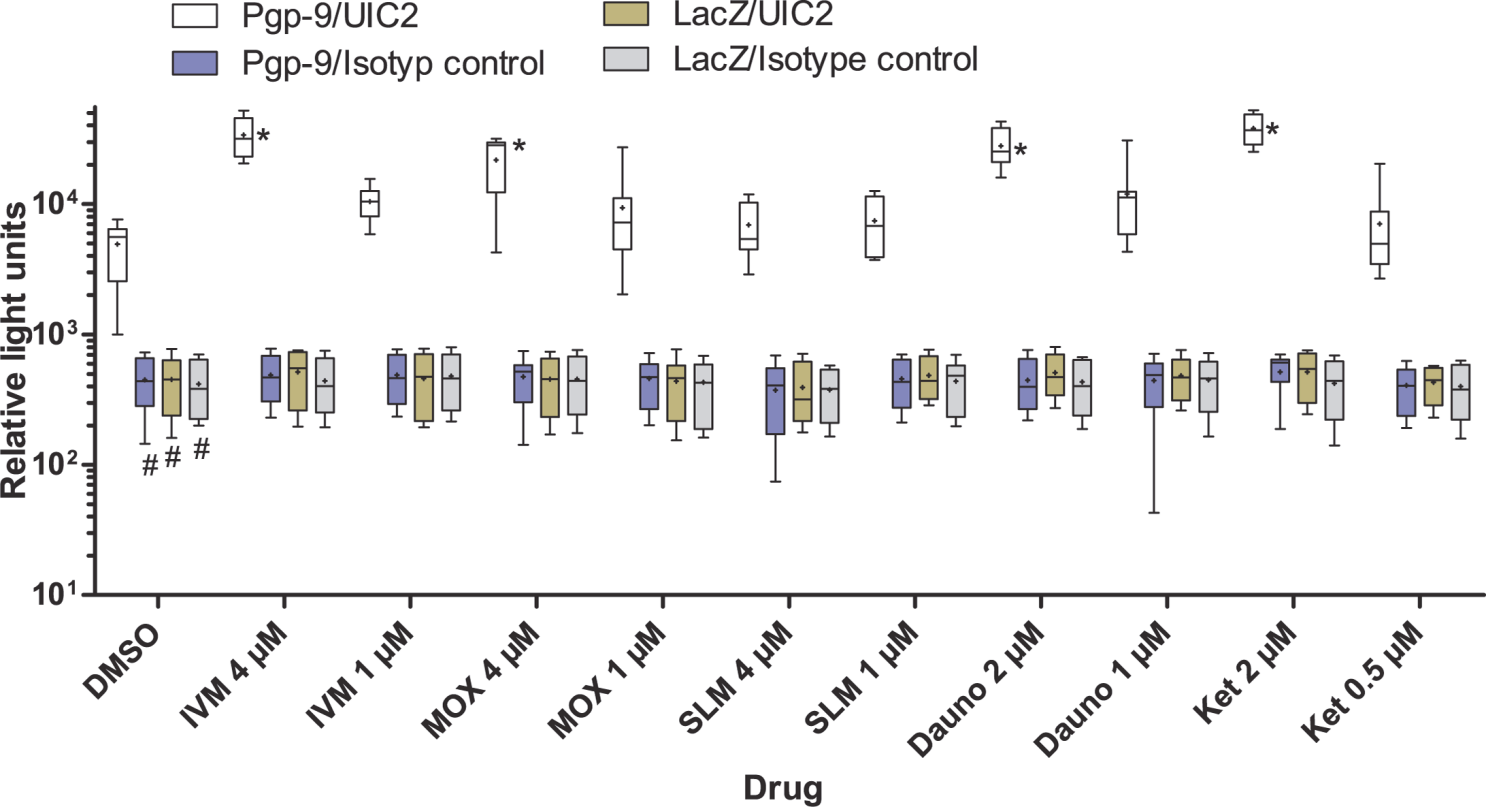
Changes in binding of UIC2 to Pgp-9 in response to the presence of drugs. AD1-7*Ceg*Pgp-9V5His or control AD1-7*lacZ* yeast cells were incubated with the indicated drugs in the presence of 1% DMSO or with DMSO alone. As an isotype control, the monoclonal antibody Mg2a-53 was used. Binding of antibodies was detected using a horseradish-peroxidase labeled secondary antibody and a luminol-based chemiluminescence reaction. Results represent 7–8 individual values recorded in 4 independent experiments with two technical replicates. Three replicates were excluded since apparently no luminol solution had been added to the wells. Whiskers represent the range of relative light units. The group means are indicated by +. Significant differences were detected using a Kruskal-Wallis test followed by a Bonferroni post hoc test against AD1-7*Ceg*Pgp-9V5His with UIC2. One test was conducted comparing the samples exposed only to DMSO (first group) representing different combinations of yeast strain and primary antibody (#, p<0.05). A second test compared the effects of different drugs on binding of UIC2 to AD1-7Pgp-9 (black symbols) (*, p<0.05).

AD1-7*Ceg*Pgp-9V5His cells showed significantly higher binding of UIC2 than of the isotype control antibody (Fig 5). Binding of either antibody to AD1-7*lacZ* was comparable to binding of the isotype control to AD1-7Pgp-9V5His and significantly lower than the binding of UIC2 to AD1-7*Ceg*Pgp-9V5His (p<0.05). In the presence of drugs, changes were observed only for the combination of AD1-7*Ceg*Pgp-9V5His with UIC2 but not with any of the combinations involving the isotype control antibody or the AD1-7*lacZ* yeasts. The higher concentrations of IVM, MOX, daunorubicin (4 μM) and Ket (2 μM) clearly increased binding of UIC2 to AD1-7*Ceg*Pgp-9V5His as demonstrated in terms of chemiluminescence intensity by roughly 5–6 fold (p<0.05), although a high variation was observed between samples (Fig 5). In contrast, the higher concentration of SLM (4 μM) had no significant effect on binding of UIC2 to AD1-7*Ceg*Pgp-9V5His cells. The lower concentrations of the drugs resulted in 0.9–1.8 fold changes in median relative light units and none of these changes was significant.

## Discussion

MLs represent the most important antiparasitic drug class with numerous fields of application in human and veterinary parasitology. Unfortunately, during the past decade their unprecedented efficacy is facing increasing resistance problems most often observed in parasitic nematodes of ruminants and horses. Non-specific mechanisms such as drug metabolism and in particular drug extrusion have been frequently implicated in anthelmintic resistance, in particular for the MLs [48] which are well established substrates of mammalian Pgps [49].

Several studies have suggested correlation between Pgp expression or genotype and ML resistance [9,50]. Particularly, Pgp-2, Pgp-9 and Pgp-11 have been implicated in ML resistance in *H*. *contortus*, *Teladorsagia circumcincta* and *P*. *equorum* [27,51–54]. In *C*. *elegans*, loss of function mutants of any Pgp gene increase IVM susceptibility significantly but effects of these mutations were in general modest to low. This is presumably due to substantial redundancy caused by 14 Pgp transporters encoded in the *C*. *elegans* genome which probably have substantial overlaps in their substrate specificity [29–31]. Effects of a putative loss of function mutation in *C*. *elegans pgp-9* were moderate in a development assay and increased IVM susceptibility by approximately 2.8-fold in terms of the EC_50_ [29].

Despite the fact that many studies dealt with the role of Pgps in anthelmintic resistance, to the best knowledge of the authors recombinant expression of only one *C*. *elegans* Pgp (Pgp-1) [55] and one parasitic nematode Pgp have been reported yet [54]. The latter, very recently published study was conducted largely in parallel with the current study published here [56] and describes expression of *H*. *contortus* Pgp-2 in porcine LLC-PK1 cells [54] known to have low endogenous transporter activity.

Detection of *Ceg*Pgp-9 by Western blotting failed although the β-galactosidase in the control strain was detectable. This could suggest that no or only very low amounts of *Ceg*Pgp-9 were produced by the cells. To evaluate *Ceg*Pgp-9 expression further, FACS analysis employing the monoclonal antibody UIC2 was initially used as an alternative detection method. This antibody was frequently used to characterize mammalian Pgps and is known to bind to a non-continuous, conformation-sensitive epitope that is only present on Pgps during certain stages of active transport/ATP hydrolysis [57,58]. Binding of UIC2 to Pgp—in particular in the presence of low concentrations of Pgp substrates—is well known to inhibit Pgp-mediated transport and the presence of Pgp substrates increases binding of UIC2 [57,59]. This antibody has also several times been used to characterize nematode Pgps by flow cytometry [32,42,60,61] and it was suggested that the epitope recognized is conserved between mammals and nematodes. The results presented here using yeasts expressing transgenic *Ceg*Pgp-9 show for the first time formally that UIC2 binds to an epitope that is conserved between nematode and vertebrate Pgps and therefore corroborate the previously published reports regarding nematode Pgps. They also clearly proof that expression of *Ceg*Pgp-9 was successful, which is further confirmed by consistent shifts in the drug concentration response curves towards higher drug concentrations.

In the Pgp-9 transgenic yeasts it was not surprising that only a minor percentage of yeast cells exposed the UIC2 epitope. It has been found previously that in the absence of any Pgp substrate—as in the FACS experiments described here—only a small fraction of mammalian cancer cells expressing Pgp appeared to be positive for UIC2 binding [59]. The frequency of positive cells was described to increase markedly in the presence of Pgp substrates. This probably explains the low frequency of UIC2-positive yeast cells observed herein, despite the usage of a clonal expression system. Nevertheless, it remains unclear why expression of the UIC2 epitope was much higher in the larger cell population. Not unexpectedly, background fluorescence (i.e. autofluorescence plus binding of the isotype control) was much higher in the larger cells from region R2. Since increased background should in principle decrease the ability to detect specific binding, the observed higher frequency of UIC2 binding despite increased background is most likely specific. The authors currently have no experimentally substantiated explanation why the expression of an epitope restricted to active Pgps is preferentially observed on large yeast cells. There are no hints in the literature that the gal-1 promoter used in the expression vector displays a cell cycle specific expression pattern. In fact, this promoter is widely used for high-level expression and cell-cycle dependency would probably counteract this aim. Increased production of endogenous Pgp substrates in the later parts of the cell cycle resulting in more active Pgp on the cell surface would be a possible explanation for the data but further experiments are required to fully understand this observation. In the chemiluminescence assay, increased binding of UIC2 but not of the isotype control antibody was observed in the presence of drugs for AD1-7Pgp-9 but not for AD1-7lacZ. Results were overall very similar to those observed in the growth assay but variation between samples was much larger. Increased binding was concentration dependent with the lower drug concentrations showing no significant effects.

The results obtained with various well characterized fungicidal substances known to be substrates of mammalian Pgps clearly show that the direct growth assay is able to evaluate the substrate specificity of transporters as previously shown by others [46] who tested interaction of cytotoxic drugs with variants of human ABCB1 in similar yeast assays. The anthelmintic drug TBZ also has fungicidal activity and could therefore be tested in the direct growth assay. Despite the fact that Pgps were recently implicated in resistance to TBZ [62], no protective effects of *Ceg*Pgp-9 were detected here. This might be explained by the large number of Pgp genes encoded in the genomes of nematodes and maybe also by species or even isolate-specific differences. Although the *Ceg*Pgp-9 analyzed in the present study does not have the potential to confer TBZ resistance, other members of the gene family might still have this capability. Moreover, the ability of Pgp-9 from other parasite species or even other *C*. *elongatus* isolates can also not be excluded from the negative data obtained in the present study. Recombinant expression of *C*. *elegans* Pgp-1 increased resistance of *Spodoptera frugiperda* Sf9 cells to the cytotoxic drugs actinomycin D and paclitaxel. ATPase activity was also stimulated by both drugs and additionally by valinomycin, progesterone and dipyridamole. In contrast many other drugs stimulated ATPase activity only marginally (e.g. vinblastine) or not at all (including verapamil and daunorubicin) [55]. Comparison with the result of the direct yeast growth assay for *Ceg*Pgp-9 suggest partially overlapping substrate spectra but also important discrepancies. In order to obtain a more complete overview of nematode Pgp substrate specificity, comparisons of all paralogs from a single nematode species with the same method would be extremely valuable.

In contrast to the BZs, most other anthelmintic and in particular most nematocidal drug classes target neuronal receptors and therefore any additionally observed fungicidal activities most likely are due to completely different modes of action. For MLs, activity against the microorganisms *Mycobacterium tuberculosis* and *Chlamydia trachomatis* has previously been described [63,64], although IVM effects against *M*. *tuberculosis* could not be confirmed by another group [65]. In *Candida* spp., milbemycin oximes have been shown to not only potentiate azole effects by inhibition of azole export but also to cause formation of reactive oxygen species and activate transcription of several genes involved in stress response pathways [66]. However, activity of MLs against AD1-7 yeasts was only observed at high concentrations and only for IVM and EPM. Since the direct growth assays can only be conducted for drugs exhibiting considerable fungicidal activity, an indirect growth assay was developed to test for interaction of MLs with susceptibility to Ket in the presence or absence of *Ceg*Pgp-9. In this assay, IVM and EPM showed strong, MOX intermediate and SLM and DRM no interaction with Ket in *Ceg*Pgp-9 transgenic AD1-7 yeasts. A similar ranking was also observed in the chemiluminescence assay were strongly increased binding of UIC2 was detected for IVM and MOX but no increase occurred in the presence of SLM. Although the growth assay was designed similarly to competitive inhibition assays with a fixed Ket concentration together with increasing ML concentrations, the observed effects do not necessarily reflect competitive inhibition. Interaction depends on the presence of *Ceg*Pgp-9 and therefore most likely on interference with Ket transport, but other types of inhibition mechanisms than competitive cannot be excluded currently. Despite this ambiguity, the assay is clearly able to identify differences between MLs regarding their interaction with *Ceg*Pgp-9 in the context of Ket. These positive interactions for example substantially corroborate previous correlative support for involvement of Pgp-9 in IVM resistance in *T*. *circumcincta* [52].

Mammalian Pgps are well known to transport several MLs and the distribution and bioavailability of MLs in different organs and tissues of the hosts are strongly influenced by the host Pgps *in vivo* [67]. Particularly IVM and DRM are known to exert strong neurotoxic effects in *mdr-1* (ABCB1a) knock-out mice and in certain dogs which have a loss of function mutation in the *mdr-1* gene. Uptake of IVM, DRM and SLM in the brain of mice is strongly decreased by Pgp transport, whereas differences are smaller for MOX and EPM [68,69]. Using cells expressing human or canine Pgp, strong interaction of IVM, EPM and DRM and weak interaction of MOX were reported [70,71]. For SLM, results of these studies were inconsistent with one [71] describing strong and the other [70] only weak interaction. In the present study, strong interaction for *Ceg*Pgp-9 with IVM, EPM and slightly weaker interactions with MOX were found to be similar to the above-mentioned observation for the mammalian orthologs. In contrast, results for DRM and SLM showed profound discrepancies between mammalian Pgp and *Ceg*Pgp-9. Future experiments comparing these transporters in the same environment are required to ultimately identify these differences as related to the transporter and not to the experimental system. Previously published results of functional analyses of nematode Pgps surprisingly describe stimulatory effects of anthelmintic drugs on extrusion of the Pgp substrate rhodamine 123 from *H*. *contortus* eggs [32]. This study also found no significant interaction of TBZ with Pgps on the nematode egg surface which is in agreement with previous results regarding mammalian Pgps [72]. For the MLs, strong effects were observed for emamectin and DRM, medium effects for abamectin, EPM and MOX, only small effects of SLM and surprisingly no significant effects of IVM, which is a very good substrate of mammalian Pgps [72] and also showed strong interaction with *Ceg*Pgp-9 here and *H*. *contortus* Pgp-2 [54]. However, the experimental approaches used here differ significantly from those used with *H*. *contortus* eggs. In contrast to stimulation of rhodamine transport by MLs for nematode Pgps [32], MLs have been consistently described to inhibit rhodamine transport by mammalian Pgps (ABCB1) [49,70–74] or by ABC transporters in azole-resistant *Candida* spp. fungi [66,75]. The effects of MLs on rhodamine transport in *H*. *contortus* eggs require further investigations to confirm these results and identify potential reasons for the observed differences. Using transfected LLC-PK1 cells, Godoy et al. [54] could recently show that MLs inhibit transport of the Pgp substrates rhodamine 123 and calcein-AM by recombinant *H*. *contortus* Pgp-2 in a concentration dependent manner. Effects of MLs differed between the assays. In the presence of rhodamine abamectin showed the strongest, IVM an intermediate and MOX the smallest influence. In contrast, effects of the MLs on transport of calcein-AM were very similar. One possible reason for the very unusual stimulation of rhodamine transport by MLs in the eggs might be the localization of Pgps in or on the eggshell in contrast to its normal localization in a plasma membrane. The drug binding site of Pgps is very complex and for mouse ABCB1b and *C*. *elegans* Pgp-1 crystal structures have been obtained by x-ray diffraction [55,76,77]. Both structures reveal that drug entry apparently occurs at the level of the inner leaflet of the plasma membrane. The localization of the substrate binding pockets in the membrane causes a significant impact of the local lipid environment on binding affinity. Indeed, binding affinity of drugs to Pgps is much higher in a lipid bilayer than in detergent micelles [55]. Lipid dependency of substrate affinity might result in changes in substrate affinity in recombinant expression systems in comparison to the natural situation. This potential systematic error can currently not be excluded for any of the systems used to express Pgps. Lipid composition of mammalian [54,70,72], insect [55] and yeast ([46] and this study) expression systems for Pgps is probably always significantly different from that of parasitic nematodes. One could propose that *C*. *elegans* would be a better expression system due to its closer phylogenetic relationship but evidence for this hypothesis is lacking. This is probably valid for soluble proteins but regarding lipid composition of plasma membranes, considerable differences between *C*. *elegans* and parasitic nematodes must be assumed, since optimal environmental temperatures of 20°C and 37°C surely imply different lipid composition to achieve similar membrane fluidities.

The mouse Pgp crystal structures in the presence of different drugs indicate drug-dependent interaction with specific amino acid residues suggesting that different, only partially overlapping binding sites for different drugs are used [76,77]. Earlier, much simpler models of mammalian Pgps considered three distinct substrate binding sites. Transport of substances binding to the R site (e.g. rhodamine 123, daunorubicin and vinblastine) is stimulated by drugs binding to the H site (e.g. Hoechst 33342 and colchicine) [78]. In contrast, Pgp substrates such as Ket and verapamil are well known to inhibit transport of other substrates in a more or less competitive manner [79].

Although nematode Pgps have been implicated not only in resistance to MLs but also to BZs [62] and have been shown to be activated by levamisole [32], current knowledge about substrate specificity of individual nematode and in particular parasitic nematode Pgps regarding anthelmintics is still very sparse. Only very recently, the brain penetration of emodepside has been shown to be increased in *mdr-1* (ABCB1a) deficient mice [80] suggesting that the cyclooctadepsipeptides [81] might also be Pgp substrates. The study presented here aims to initiate more systematic approaches, leading to detailed comparison of substrate spectra. Such comparisons could be made between (i) Pgp paralogs within a single species, (ii) Pgp orthologs from different species and (iii) alleles of individual Pgp genes from independent isolates of the same parasite species with different resistance status. This aim is currently hampered by several problems. First, at least 10 Pgp genes can be suspected to be encoded in the genomes of most parasitic nematodes with no complete collection of full-length sequences available for any species. Secondly, a large number of anthelmintic drugs must be considered to be potential Pgp substrates. Finally, there are huge differences between drugs belonging to the same drug class regarding interaction with the same Pgp. Therefore, research aiming to provide a detailed picture of interaction of anthelmintic drugs with individual Pgps is obviously still in its infancy.

In conclusion, this report shows functional interaction of MLs with a Pgp from a target species. At least for Pgp-9 of *C*. *elongatus* it was clearly shown, that there are marked differences regarding interaction of individual MLs with this particular transporter. The growth assay described herein is a relatively simple and inexpensive method for the investigation of the functional analysis of transport by Pgps of parasitic nematodes but potentially also arthropods. In comparison to the chemiluminescence-based UIC2 binding assay, which suffers from very high hands-on times and only limited numbers of samples that can be analyzed in parallel, and to the assay using stably transfected mammalian cells [54], the yeast assay described here is cheap, largely automatically performed and even has the potential to be up-scaled to 384 well plates. The chemiluminescent UIC-binding assay offers the advantage that it does not rely on interaction of two drugs. The transport assays using mammalian cells described by Godoy et al. [54] has revealed that MLs differ in interaction with *H*. *contortus* Pgp-2 depending on the co-substrate that is used to show increased intracellular accumulation (rhodamine 123 vs. calcein-AM). The same does most likely also apply to the growth assay, i.e. using another fungicidal drug than Ket might result in another ranking of MLs. Since numerous Pgp genes as well as alleles from the same Pgp gene have been described for different parasitic nematodes, this new assay provides the opportunity to directly compare their substrate specificity and their ability to interact with anthelmintics. Therefore, this approach provides a powerful tool to compare substrate specificity of different Pgps, enabling pharmacological and functional research in the future, including various anthelmintic drug classes and compounds. Due to the episomal expression system, Pgps differing only in single amino acid changes can be expected to be expressed at equal levels as previously exploited to analyze human Pgp variants [46]. In mammalian systems, targeted integration would be required for such comparisons. Usage of targeted integration systems such as homologous or targeted integration is also much easier in the yeast than in the mammalian system. This should also allow determination of effects of non-synonymous SNPs in Pgps that were described to be associated with ML resistance. Finally, the specific functional analysis of Pgps as modulators of anthelmintic activity will contribute to the understanding of AR mechanisms, foster the development of tools for early detection of resistance and increase our ability to prevent or postpone the development of AR.

## Supporting Information

S1 Table Primers used for amplification of *Cylicocyclus elongatus* Pgp-9. (PDF)

S2 Table NCBI accession numbers and Wormbase ID for protein sequences used for maximum likelihood tree. (PDF)

S1 Fig Expression analysis of AD1-7*Ceg*Pgp-9 (lanes 1 and 2) and AD1-7*Ceg*Pgp-9V5His (lanes 3 and 4) yeast cells. Lanes 2 and 4 are controls without reverse transcription. M, 100 bp marker.
(PDF)

S2 Fig Western blot analysis of yeast cells expressing CegPgp-9V5His (left) and lacZ (right). Proteins were separated by SDS PAGE and transferred to nitrocellulose membranes. Detection of recombinant proteins was conducted with an anti-V5 monoclonal antibody.
(PDF)

S3 Fig Representative growth curves for AD1-7*Ceg*Pgp-9. The OD_600_ was automatically recorded every 10 min over a period of 48 h. The negative control contained only the vehicle (1% DMSO).
(PDF)

S4 Fig Effects of doramectin (DRM) on susceptibility to ketoconazole (Ket). Black symbols show relative growth in the presence and blue symbols in the absence of Ket. Ket concentrations were 0.72 μM for AD1-7*Peg*V5His and 0.18 μM for AD1-7*lacZ*. The yeast strains AD1-7*Peg*V5His (*A*) and AD1-7*lacZ* (*B*) were compared for effects of DRM. *, p < 0.05 vs. the same drug concentration in the absence of Ket using a two way ANOVA.
(PDF)
