# Supplementary material for: Macrocyclic Lactones Differ in Interaction with Recombinant P-Glycoprotein 9 of the Parasitic Nematode Cylicocylus elongatus and Ketoconazole in a Yeast Growth Assay
Source: PLoS Pathog. 2015 Apr 7;11(4):e1004781. doi: 10.1371/journal.ppat.1004781 (PMC4388562; doi:10.1371/journal.ppat.1004781)
Supplement: S1 Table — (PDF) [file ppat.1004781.s001.pdf]

Table S1 Primers used for amplification of *Cylicocyclus elongatus* Pgp-9

Primer	Sequence (5′-3′)	T_a	Polymerase
Ceg-Pgp9-vorF2	CTMACNCTNATYATGATGTC	50/60 °C	Accu Prime[a]
Ceg-Pgp9-3R1	GCGCTCATTAGAAATCCTAAAATTC	55 °C	Accu Prime[a]
Ceg-Pgp9-3R2	AATCCTAAAATTCTTCTGCTTGATG	55 °C	Accu Prime[a]
Ceg-Pgp9-5R1	CCGCCAAAGCAACGTCGTATCTT	-	cDNA synthesis
Ceg-Pgp9-5R2	GGTTGGCCATTGAAGGCAATGACTGTCCTCATT	60 °C	Accu Prime[a]
Ceg-Pgp9-5R3	TCCAAAGTAGAATGCGATACCAATACCACAACAT A	50/60 °C	Accu Prime[a]
Ceg-Pgp9-full-up	GAGGTAGTCGCATAAATGGGACTGTTCAA	68 °C	Phusion[b]
Ceg-Pgp9-full-lo	TTGCTTACACGTATCGAGATCATAG	68 °C	Phusion[b]
pYes-Ceg-Pgp9-start	AAAAAAATGGGACTGTTCAAAAAGAAAGAAGAA	55 °C	Accu Prime HF[c]
pYes-Ceg-Pgp9-lo	AAGATCTAAAGTCAGTTGGTGTGAATATTC	55 °C	Accu Prime HF[c]
pYes-Ceg-Pgp9-His	GTTGGTGTGAATATTCTGTTTCTGCGTGAG	55 °C	Accu Prime HF[c]
Oligo d(T)-anchor primer	GACCACGCGTATCGATGTCGACTTTTTTTTV	55/60 °C	Accu Prime[a]
PCR anchor primer	GACCACGCGTATCGATGTCGAC	55/60 °C	Accu Prime[a]

[a]Accu Prime™ Taq DNA Polymerase
[b]Phusion™ Hot Start Polymerase
[c]Accu Prime™ High Fidelity Polymerase
