# Supplementary material for: Macrocyclic Lactones Differ in Interaction with Recombinant P-Glycoprotein 9 of the Parasitic Nematode Cylicocylus elongatus and Ketoconazole in a Yeast Growth Assay
Source: PLoS Pathog. 2015 Apr 7;11(4):e1004781. doi: 10.1371/journal.ppat.1004781 (PMC4388562; doi:10.1371/journal.ppat.1004781)
Supplement: S2 Table — (PDF) [file ppat.1004781.s002.pdf]

Table S2 NCBI accession numbers and Worm Base ID for protein sequences used for maximum likelihood tree.

Name	Organism	GenBank accession no.	WormBase ID
*Asu*MRP-1	*Ascaris suum*	ADY40620	
*Asu*MRP-3a	*Ascaris suum*	ADY40644	
*Asu*MRP-3b	*Ascaris suum*	ADY40573	
*Bma*MRP-3	*Brugia malayi*	XP_001896434	
*Bma*Pgp-1	*Brugia malayi*	XP_001900095	
*Bma*Pgp-2	*Brugia malayi*	XP_001897744	
*Cbr*Pgp-1	*Caenorhabditis briggsae*	XP_002634978	BP:CBP17817
*Cbr*Pgp-2	*Caenorhabditis briggsae*	XP_002639421	BP:CBP33028
*Cbr*Pgp-3	*Caenorhabditis briggsae*	XP_002643979	BP:CBP26138
*Cbr*Pgp-4	*Caenorhabditis briggsae*	XP_002643978	BP:CBP2677
*Cbr*Pgp-7	*Caenorhabditis briggsae*	XP_002644094	BP:CBP25824
*Cbr*Pgp-8	*Caenorhabditis briggsae*	XP_002644095	BP:CBP34515
*Cbr*Pgp-9	*Caenorhabditis briggsae*	XP_002638613	BP:CBP25885
*Cbr*Pgp-10	*Caenorhabditis briggsae*	XP_003117716	BP:CBP25360
*Cbr*Pgp-11	*Caenorhabditis briggsae*	XP_002629994	BP:CBP09377
*Cbr*Pgp-12	*Caenorhabditis briggsae*	XP_002645224	BP:CBP35051
*Cbr*Pgp-13	*Caenorhabditis briggsae*	XP_002645222	BP:CBP27772
*Cbr*Pgp-14	*Caenorhabditis briggsae*	XP_002645220	BP:CBP35857
*Cbr*Pgp-CBG12969	*Caenorhabditis briggsae*	XP_002630530	BP:CBP17602
*Cel*Pgp-1	*Caenorhabditis elegans*	NP_502413	WP:CE11932
*Cel*Pgp-2	*Caenorhabditis elegans*	NP_491707	WP:CE41207
*Cel*Pgp-3	*Caenorhabditis elegans*	NP_509901	WP:CE03818
*Cel*Pgp-4	*Caenorhabditis elegans*	NP_001257143	WP:CE03308
*Cel*Pgp-5	*Caenorhabditis elegans*	NP_001257116	WP:CE43003
*Cel*Pgp-6	*Caenorhabditis elegans*	NP_001041287	WP:CE40818
*Cel*Pgp-7	*Caenorhabditis elegans*	NP_509812	WP:CE36668
*Cel*Pgp-8	*Caenorhabditis elegans*	NP_509811	WP:CE31624
*Cel*Pgp-9	*Caenorhabditis elegans*	NP_507487	WP:CE15714
*Cel*Pgp-10	*Caenorhabditis elegans*	NP_509205	WP:CE40807
*Cel*Pgp-11	*Caenorhabditis elegans*	NP_495674	WP:CE34788
*Cel*Pgp-12	*Caenorhabditis elegans*	NP_510126	WP:CE03260
*Cel*Pgp-13	*Caenorhabditis elegans*	NP_510127	WP:CE40253
*Cel*Pgp-14	*Caenorhabditis elegans*	NP_510128	WP:CE0262
*Con*Pgp-2	*Cooperia oncophora*	AGJ71178	
*Con*Pgp-3	*Cooperia oncophora*	AGJ71177	
CegPgp-11	*Cylicocyclus elongatus*	KJ701410	
*Dme*MDR-50	*Drosophila melanogaster*	AAA16186	
*Dme*MDR-49	*Drosophila melanogaster*	AAA28679	
*Dme*MDR-65	*Drosophila melanogaster*	AAA28680	
*Hco*Pgp-2	*Haemonchus contortus*	AAC38987	
*Mmu*ABCB-1b	*Mus musculus*	NP_035205	
*Mmu*Pgp	*Mus musculus*	AAA39514	
*Mca*Pgp-L	*Mytilus californianus*	ABS83556	
*Mga*Pgp-L	*Mytilus galloprovincialis*	CAX46411	
*Ovo*Pgp-1	*Onchocerca volvulus*	AAD49436	
*Ovo*Pgp	*Onchocerca volvulus*	AAX82635	
*Ovo*Pgp-L	*Onchocerca volvulus*	AAD49563	
*Peq*Pgp-11	*Parascaris equorum*	JX308230	
*Peq*Pgp-16	*Parascaris equorum*	JX308231	
*Phu*MRP-1	*Pediculus humanus corporis*	XP_002425149	
*Phu*MRP-2	*Pediculus humanus corporis*	XP_002432260	
*Phu*MRP-3	*Pediculus humanus corporis*	XP_002426586	
*Phu*MRP-4	*Pediculus humanus corporis*	XP_002425021	
*Ppa*Pgp-1	*Pristionchus pacificus*		PP:PP30697
*Ppa*Pgp-9	*Pristionchus pacificus*		PP:PP30465
*Sma*Pgp-1,2,3	*Schistosoma mansoni*	XP_002574196	
