# Supplementary figures and images for: Macrocyclic Lactones Differ in Interaction with Recombinant P-Glycoprotein 9 of the Parasitic Nematode Cylicocylus elongatus and Ketoconazole in a Yeast Growth Assay
Source: PLoS Pathog. 2015 Apr 7;11(4):e1004781. doi: 10.1371/journal.ppat.1004781 (PMC4388562; doi:10.1371/journal.ppat.1004781)

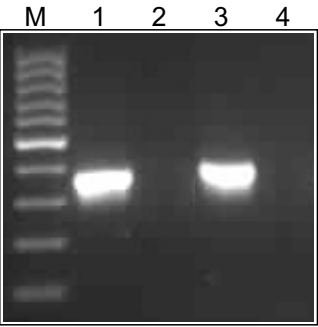

Supplement: S1 Fig — Lanes 2 and 4 are controls without reverse transcription. M, 100 bp marker. (PDF) [file ppat.1004781.s003.pdf]

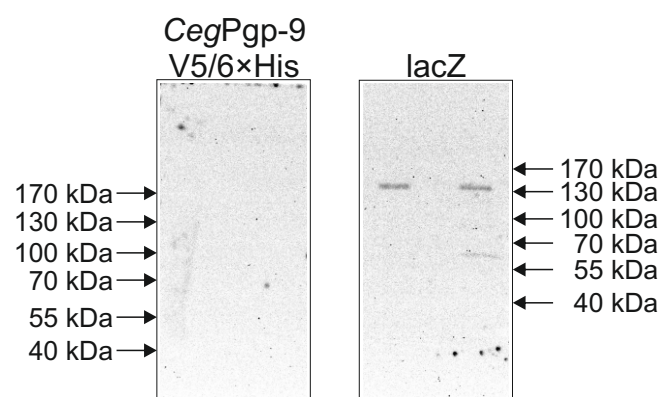

Supplement: S2 Fig — Proteins were separated by SDS PAGE and transferred to nitrocellulose membranes. Detection of recombinant proteins was conducted with an anti-V5 monoclonal antibody. (PDF) [file ppat.1004781.s004.pdf]

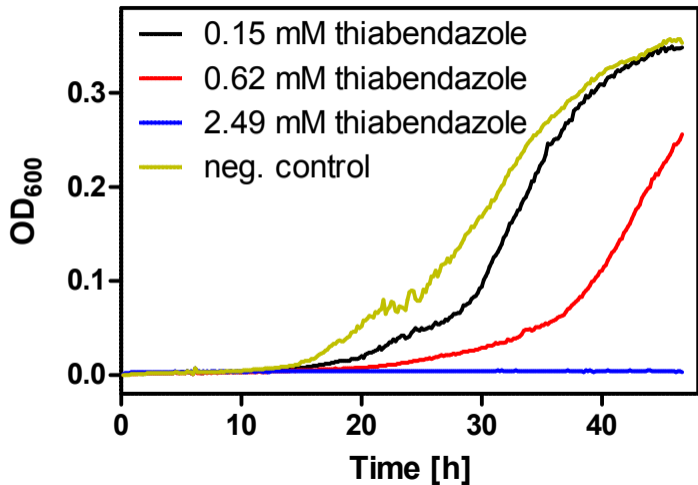

Supplement: S3 Fig — The OD600 was automatically recorded every 10 min over a period of 48 h. The negative control contained only the vehicle (1% DMSO). (PDF) [file ppat.1004781.s005.pdf]

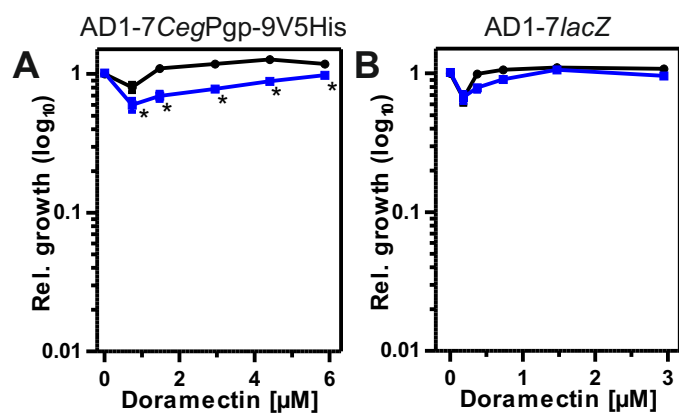

Supplement: S4 Fig — Black symbols show relative growth in the presence and blue symbols in the absence of Ket. Ket concentrations were 0.72 μM for AD1-7PegV5His and 0.18 μM for AD1-7lacZ. The yeast strains AD1-7PegV5His (A) and AD1-7lacZ (B) were compared for effects of DRM. *, p < 0.05 vs. the same drug concentration in the absence of Ket using a two way ANOVA. (PDF) [file ppat.1004781.s006.pdf]
